# Anaphylactic reaction due to razor shell

**DOI:** 10.1186/2045-7022-1-S1-P57

**Published:** 2011-08-12

**Authors:** Victoria Cardona, Lorena Soto, Moisés Labrador, Olga Luengo

**Affiliations:** 1 Hospital Universitari Vall d′Hebron, Allergology, Barcelona, Spain

## Background

a 36-year-old woman, with a history of childhood asthma allergic to house dust mites, noticed one year prior to her arrival at our unit an episode of generalized itching, dyspnea, severe rhinoconjunctivitis and abdominal pain after eating rice with razor shell. After that, she had tolerated rice alone. Razor shell (Ensis ensis) is a common mollusc, which has an elongated fragile and narrow shell shaped like a cut-throat razor.

## Methods

Skin prick testing (SPT) with commercial standard food extracts and prick-to-prick testing with razor shell, ImmunoCAP ISAC (Phadia) determination as well as protein separation (SDS-PAGE) and immunobloting analyses were carried out.

## Results

SPT with standard commercial food extracts including shellfish extract (clam, mussel, octopus, shrimp) and Anisakis simplex were all negative. Prick-to-prick testing with raw and boiled razor shell resulted positive. ISAC® determination was negative for all tropomyosin indicators as well as other food allergens. IgE immunobloting performed with the serum of the patient over razor shell extract SDS – PAGE separation revealed four major bands at 40-45 kDa and 70-80 kDa.

## Conclusion

We describe a case of anaphylaxis caused by selective sensitization razor shell. To our knowledge, there are only two other cases of immediate hypersensitivity to razor shell published to date. In our study we highlight the identification of band profiles different to those identified in previous reports, which were present both in raw and boiled extract.

